# Metagenomic next-generation sequencing of aqueous humor guides targeted therapy for *Staphylococcus epidermidis* endophthalmitis in chronic ocular graft-versus-host disease: a case report

**DOI:** 10.3389/fimmu.2026.1966692

**Published:** 2026-10-19

**Authors:** Jingjing Wang, Junyu Hu, Xing Liang, Fang Yuan, Lijuan Ren, Tao Wang, Zhilin Gao, Yajian Duan

**Affiliations:** 1 Third Hospital of Shanxi Medical University, Shanxi Bethune Hospital, Shanxi Academy of Medical Sciences, Tongji Shanxi Hospital, Taiyuan, China; 2 Shanxi Bethune Hospital, Shanxi Academy of Medical Sciences, Tongji Shanxi Hospital, Third Hospital of Shanxi Medical University, Taiyuan, China; 3 Tongji Hospital, Tongji Medical College, Huazhong University of Science and Technology, Wuhan, China

**Keywords:** case report, endophthalmitis, metagenomic next-generation sequencing, ocular graft-versus-host disease, *Staphylococcus epidermidis*

## Abstract

**Purpose:**

To report a case of *Staphylococcus epidermidis* endophthalmitis in chronic ocular graft-versus-host disease, refractory to empirical broad-spectrum antibiotics, rapidly identified by metagenomic next-generation sequencing of aqueous humor, and successfully managed with targeted vancomycin without vitrectomy.

**Methods:**

Case report.

**Results:**

A 35-year-old male with chronic oGVHD following allo-HSCT for acute myeloid leukemia developed endophthalmitis after 10 months of progressive disease. Slit-lamp examination revealed central corneal ulceration (4 × 2 mm) with hypopyon (1 mm). B-scan ultrasonography confirmed mild vitreous haze. Conventional microbiological workup of aqueous humor—including Gram stain, potassium hydroxide (KOH) mount, and bacterial/fungal culture—yielded negative results. Aqueous humor mNGS detected 5,543 unique reads of *S. epidermidis* (genome coverage 12.3%, no co-pathogens), exceeding the clinical significance threshold. Based on this result, therapy was switched to targeted intracameral and periocular vancomycin (10 mg/ml) combined with intravenous vancomycin (0.5 g twice daily). Vancomycin was selected for its reliable Gram-positive coverage, including coagulase-negative *staphylococci*, referencing the established intravitreal dose of 1 mg/0.1 mL. The infection was controlled without corneal perforation or vitrectomy. AMT was performed for persistent epithelial defects. At 1.5 years follow-up, anatomical preservation was maintained (BCVA 20/400 OD; 20/40 OS).

**Conclusion:**

In immunosuppressed oGVHD patients with suspected endophthalmitis, aqueous humor mNGS overcomes the diagnostic limitations of conventional culture and guides a timely switch from empirical antibiotics to targeted therapy, achieving infection control and anatomical preservation without invasive vitrectomy or enucleation.

## Background

The ocular GVHD (oGVHD) is a complication of the GVHD and can be manifested in the form of dry eyes, irritation, redness and more serious conditions including corneal ulcers and perforation. It has been estimated that 30-70% of hematopoietic stem cell transplants result in GVHD, with over half of these cases having symptoms of dry eye (1, 2). In extreme circumstances, it may even cause enucleation, corneal ulceration and perforation. oGVHD endophthalmitis is very uncommon. The combination of *S. epidermidis* endophthalmitis as a consequence of chronic oGVHD with an epithelial defect, which was not previously reported, but mNGS of aqueous humor to confirm the disease, is a new clinical case, as well as the use of mNGS to treat other ocular infections are both described separately in literature (**Table 1**). Majority of the published reports are of fungal or polymicrobial origin with the use of topical corticosteroids being long term. *S. epidermidis* is one of the most common commensals on the skin and is rarely found in the endophthalmitis and difficult to diagnose because it is a possible contaminant. A culture-free metagenomic next-generation sequencing (mNGS) test is also promising to detect pathogens from small-volume intraocular fluids samples within a short period of time. Wu et al. (3)showed that this method is much more sensitive than conventional culture (92.2%) and positivity rate (85.2%) when compared to conventional culture in immunocompromised patients after allo-HSCT, and it changed empirical diagnosis in 56.4% of the patients. This is the first documented example of the endophthalmitis caused by *S. epidermidis* in the case of chronic oGVHD confirmed by mNGS of aqueous humor.

**Table 1:** Reported cases of endophthalmitis in oGVHD patients: comparison with the present case.

Reference	Pathogen	GVHD type	Diagnostic method	Sample site	mNGS used	Vitrectomy performed	Outcome/key features
Adrean & Puklin, 2007 (4)	*S. epidermidis*	Acute GVHD	Vitreous culture + PCR	Vitreous	No	Yes (therapeutic)	Resolved (BCVA 20/50); corneal perforation led to endophthalmitis; commensal bacteria seeded vitreous
Naharwal et al., 2025 (5)	*F. oxysporum*	Acute GVHD	Vitreous biopsy culture + morphology	Vitreous	No	Yes	Poor (total RD, no vision); first F. oxysporum endogenous endophthalmitis; initially misdiagnosed as CMV retinitis
Kishida et al., 2025 (6)	*F. solani* species complex	Steroid-refractory GVHD	Blood culture + PCR (EF1α gene sequencing)	Blood	No	Yes	Enucleation; death (day 119 post-HSCT); disseminated fusariosis; VRCZ resistance (MIC>8); multi-organ failure
Current case	*S. epidermidis*	Chronic oGVHD	mNGS of aqueous humor (5,543 reads; 12.3% coverage)	Aqueous humor	Yes	No	Controlled (BCVA 20/400); FIRST mNGS-confirmed in chronic oGVHD; hypopyon + B-scan vitreous haze; AMT for persistent epithelial defect; NO vitrectomy or corneal perforation

GVHD, graft-versus-host disease; HCT, hematopoietic stem cell transplantation; mNGS, metagenomic next-generation sequencing; RD, retinal detachment; VRCZ, voriconazole; AMT, amniotic membrane transplantation; BCVA, best-corrected visual acuity.

### Case presentation

A 35-year-old man was referred to our ophthalmology department with complaints of bilateral eye redness, photophobia, dryness for 10 months. He was diagnosed with corneal epithelial defects before, and treated with artificial tears, bovine serum protein extract eye gel, tobramycin eye drops, but poor efficacy. He received allogeneic hematopoietic stem cell transplantation for acute myeloid leukemia 1.5 years ago. Currently, he was under systemic immunosuppressive therapy with prednisolone acetate 60 mg once daily, cyclosporine 75 mg twice daily. BCVA was light perception in the right eye and hand motion at 33 cm in the left eye. Right eye IOP was 31 mmHg, left eye IOP was 12 mmHg. Bilateral eyelid margin congestion, keratinization, meibomian gland orifice blockage, punctal occlusion, corneal edema, opacity, epithelial defects could be seen; iris and pupil could be vaguely seen. Considering his history of bone marrow transplantation and new-onset dry keratoconjunctivitis, Schirmer test result was 1 mm/2 mm, and the diagnosis was changed to oGVHD (**Figures 1A, B**). The patient was treated with topical prednisolone acetate eye drops, cyclosporine eye drops, and tacrolimus eye drops for immunosuppression, along with autologous serum and sodium hyaluronate eye drops to alleviate dry eye symptoms and promote corneal epithelial healing. After 10 days of treatment, the patient’s condition improved, and he was discharged with BCVA of 20/100 in the right eye and 20/40 in the left eye. Three months later, the patient returned with right eye pain and vision loss for 2 days. Examination revealed corneal ulceration and hypopyon in the right eye, raising suspicion of bacterial infection during immunosuppressive therapy. B-scan ultrasonography was performed, revealing mild vitreous haze in the right eye, In the setting of a central corneal ulcer with hypopyon, even mild vitreous opacification in an immunosuppressed host is highly suggestive of intraocular extension supporting the diagnosis of endophthalmitis rather than severe keratitis alone (**Figure 1C**). The patient was immediately hospitalized for right eye anterior chamber puncture, and aqueous humor samples were sent for metagenomic next-generation sequencing (mNGS). Prior to anterior chamber puncture, the ocular surface and periorbital skin were thoroughly disinfected with 5% povidone-iodine. Disposable sterile instruments were used, and strict aseptic protocols were followed to prevent intraocular infection. Empirical treatment with intravenous cefuroxime 1.5g daily and frequent topical antibiotic eye drops was initiated. Three days later, despite treatment, the patient’s condition worsened with corneal endothelial plaque(**Figure 1D**). Confocal microscopy showed no fungal organisms. Conventional microbiological workup of the aqueous humor—including Gram stain, potassium hydroxide (KOH) mount, and bacterial/fungal culture—yielded negative results. Metagenomic results confirmed *Staphylococcus epidermidis* infection with 5,543 unique sequence reads and genome coverage of 12.3%, with no co-pathogens identified. No fungi, viruses, or other bacterial pathogens were detected. The read count significantly exceeded the established clinical significance threshold (>100 reads with >5x coverage for bacterial pathogens (3)), and negative controls (reagent blank and sampling environment) showed no *S. epidermidis* signal, making contamination unlikely. Given the concordance between the mNGS result and the clinical phenotype (corneal ulcer with hypopyon), *S. epidermidis* was interpreted as the causative pathogen. Meanwhile, laboratory examinations including routine blood test, C-reactive protein and erythrocyte sedimentation rate were all within normal ranges, and the patient exhibited no clinical manifestations such as fever or chills, thus excluding the possibility of endogenous endophthalmitis secondary to sepsis. We promptly discontinued cyclosporine and tacrolimus eye drops, administered vancomycin injection (10 mg/ml, 0.05ml) into the vitreous cavity, anterior chamber, and periorbital region (10mg/ml, 2.5ml), combined with intravenous vancomycin infusion (0.5 g twice daily).The intravitreal vancomycin dose of 1 mg/0.1 mL follows established protocols for Gram-positive endophthalmitis (7).The treatment strategy was guided by the Endophthalmitis Vitrectomy Study (EVS) recommendations (8)and established pharmacokinetic data on intracameral vancomycin therapy: vancomycin was selected for its reliable Gram-positive coverage, including coagulase-negative *Staphylococci*, and its established safety profile for intraocular injection. Topical treatment included hourly moxifloxacin eye drops, three times daily gatifloxacin eye gel, and daily periocular vancomycin injections (10mg/ml, 2.5ml). Given that prolonged systemic corticosteroid therapy (prednisolone 60 mg daily) conferred limited additional anti-GVHD efficacy while substantially increasing infection risk, systemic steroids were tapered and discontinued in consultation with the hematology team. To maintain adequate immunosuppression for GVHD prophylaxis while reducing steroid-related infectious complications, oral mycophenolate mofetil 0.5 g twice daily was added to the existing cyclosporine regimen. After 10 days of targeted vancomycin therapy, the right corneal ulcer gradually healed and hypopyon resolved, further validating the mNGS-guided diagnosis and treatment strategy. Notably, the infection was controlled without progression to corneal perforation, obviating the need for therapeutic vitrectomy or enucleation that often accompanies advanced endophthalmitis in immunocompromised hosts. Nevertheless, persistent corneal epithelial defect and corneal bullae caused severe blepharospasm and unbearable pain (**Figure 1E**). Therefore, amniotic membrane transplantation was performed to facilitate corneal epithelial healing. AMT was performed only after infection control was achieved and was not intended as an anti-infective measure. On the first postoperative day, symptoms subsided, and the patient could open the eye freely.

**Figure 1:**
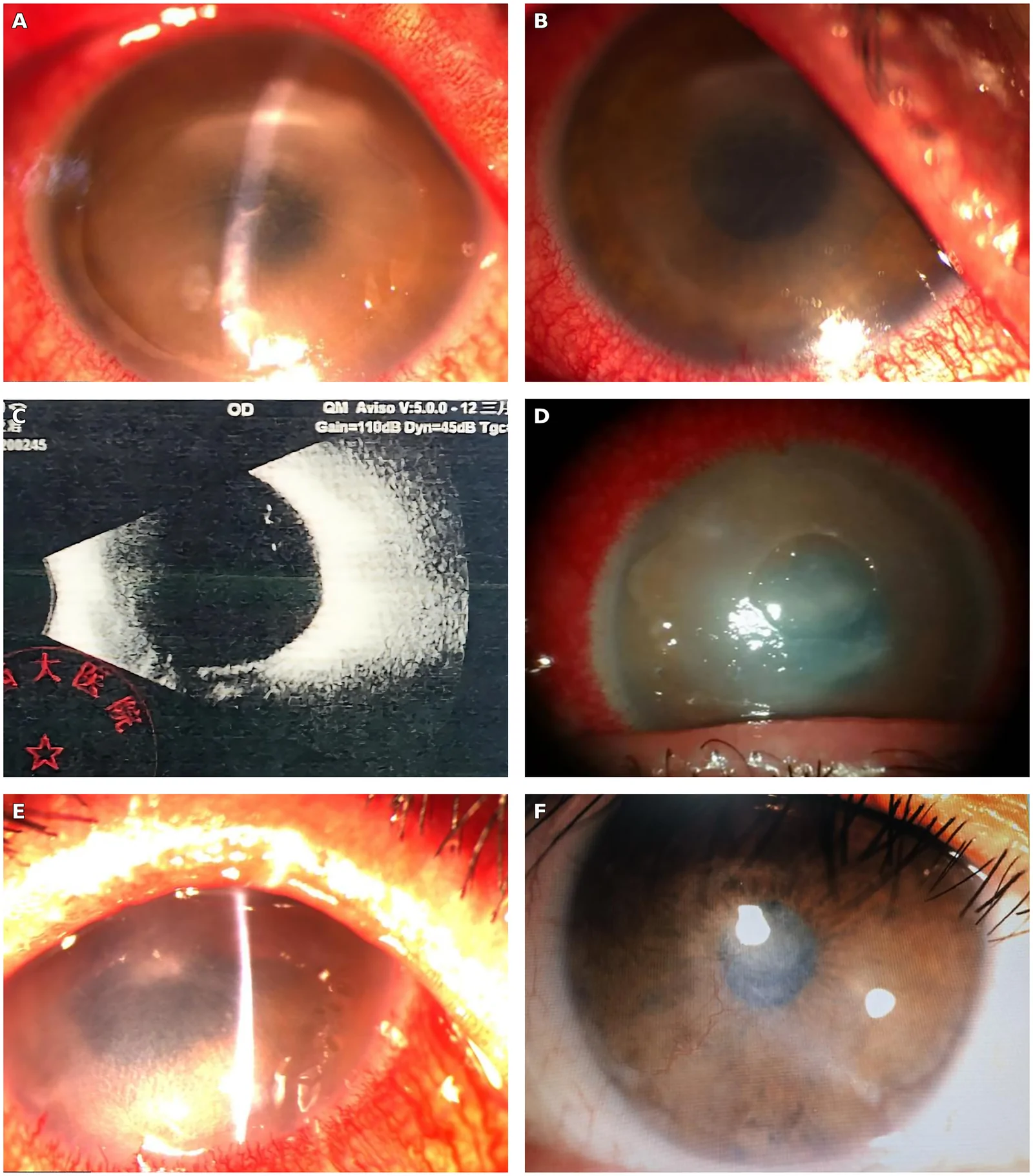
**(A)** Right eye (OD) at initial presentation showing eyelid-margin hyperemia and keratinization, atrophy of the meibomian-gland orifices, punctal occlusion, and a dry, diffusely hyperemic conjunctiva. The cornea exhibits generalized edema and opacification with a large epithelial defect and underlying stromal haze. **(B)** Left eye (OS) at initial presentation demonstrating a central, irregular epithelial defect accompanied by stromal edema; the eyelid and conjunctival findings are similar to those in the right eye. **(C)** B-scan ultrasonography of the right eye revealing mild vitreous haze. **(D)** Right eye on day 3 of the second admission showing a central stromal ulcer measuring approximately 4 × 2 mm, surrounded by marked stromal edema; a 1-mm hypopyon is faintly visible beneath the ulcer. **(E)** Right eye after 10 days of treatment showing gradual healing of the corneal ulcer, resolution of the hypopyon, and a persistent corneal epithelial defect. **(F)** Final follow-up of the right eye showing a transparent cornea with intact epithelium and a subtle neovascular pannus located inferotemporally.

Metagenomic next-generation sequencing (mNGS) was performed by Zhide Diagnostics (Beijing, China). Briefly, total nucleic acids were extracted from the aqueous humor sample, followed by high-throughput sequencing and bioinformatic analysis. DNA libraries were constructed and sequenced on an Illumina platform. Bioinformatic analysis included quality control, removal of human host sequences by alignment to the human reference genome, and subsequent alignment of remaining reads to a curated microbial database. A positive threshold of >100 unique reads with >5× genome coverage was applied for bacterial pathogens, as previously described in post-allo-HSCT immunocompromised patients (3). Negative controls included reagent blanks and sampling environment swabs.

During 1.5 years of follow-up, the patient remained asymptomatic in daily life, with discontinued systemic medications and continued topical cyclosporine eye drops twice daily and sodium hyaluronate eye drops three times daily. BCVA was 20/400 in the right eye and 20/40 in the left eye, with transparent corneas and minimal neovascularization in the right eye (**Figure 1F**). The patient demonstrated excellent adherence to the prescribed topical regimen throughout the 1.5-year follow-up period, with no reported adverse effects or treatment interruptions. The clinical timeline of this index case is presented as a flow diagram in **Figure 2**.

**Figure 2:**
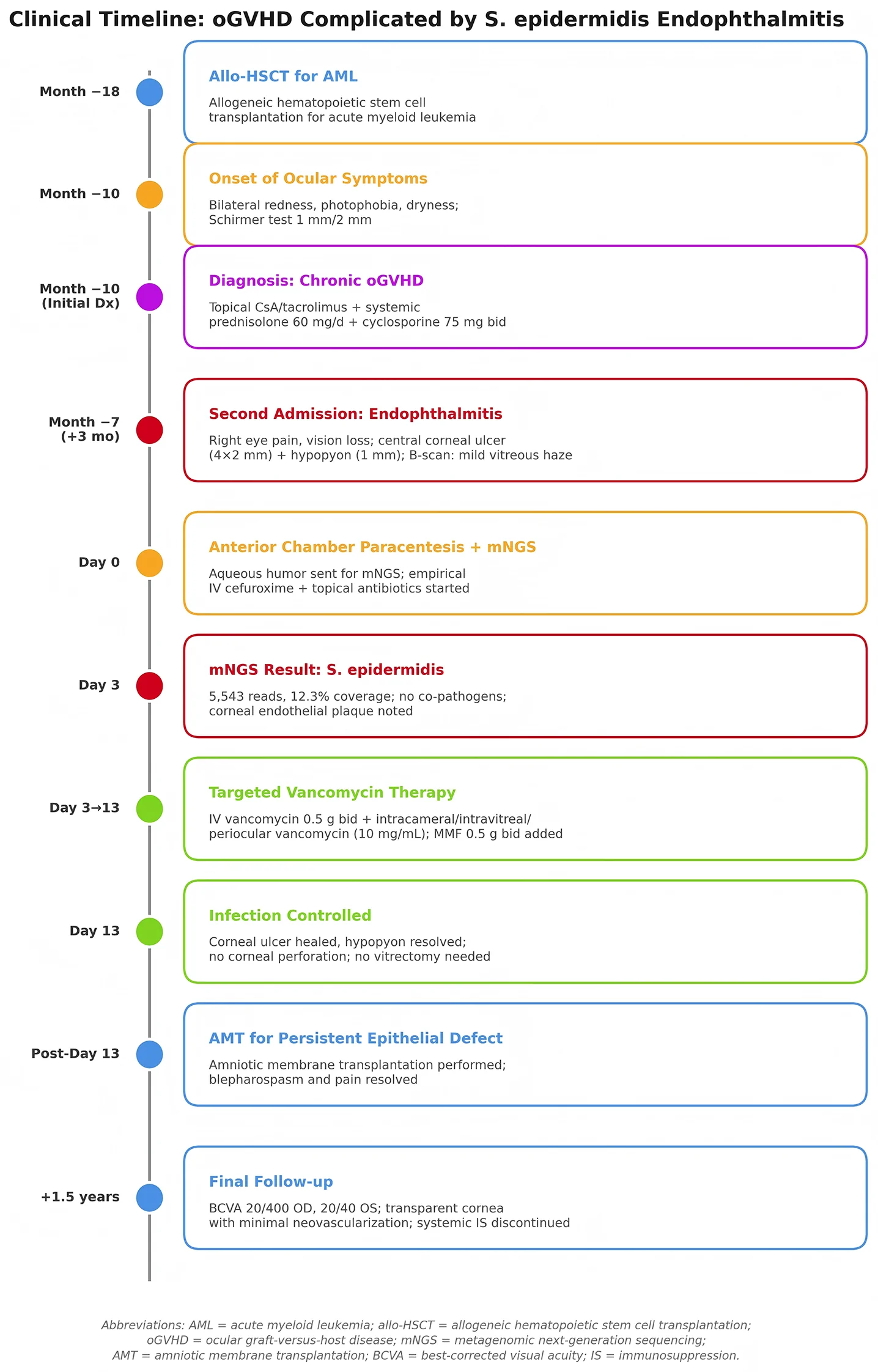
Clinical course of a patient with ocular graft-versus-host disease (oGVHD) complicated by infectious keratitis.

### Discussion

This case represents the first reported instance of *S. epidermidis* endophthalmitis in chronic oGVHD confirmed by mNGS of aqueous humor. Several aspects of this case warrant detailed discussion in the context of existing literature. The progression from epithelial defect to intraocular infection in an immunosuppressed oGVHD patient highlights a potentially underrecognized complication pathway. Early recognition of infectious complications in oGVHD is critical because chronic ocular surface inflammation and immunosuppression create a permissive environment for opportunistic pathogens. In a retrospective cohort of 118 patients with delayed-onset oGVHD, Y Liao et al. (9) demonstrated that delayed diagnosis was associated with more severe ocular manifestations, including corneal complications. In the present case, the patient had been treated only with antibiotics, artificial tears, and ocular surface repair agents for one year post-transplantation, missing the optimal window for oGVHD-specific therapy. By the time endophthalmitis developed, the meibomian glands had completely atrophied and lid margin keratinization was established. Meibomian gland imaging at the initial visit revealed near-total (grade 3) gland loss, consistent with advanced chronic oGVHD-related meibomian gland dysfunction. Early detection of MGD through meibomian gland imaging can help predict oGVHD and guide physicians to initiate appropriate treatment before significant damage occurs (10).

Long-term use of local and systemic immunosuppressants exposes oGVHD patients to a high risk of infection. Epithelial disruption is a key factor commonly observed in oGVHD individuals, arising from inflammatory cytokines and collagen-degrading enzymes that degrade the underlying extracellular matrix. As an ocular surface commensal organism, *S. epidermidis* can colonize corneal ulcers and penetrate the cornea through compromised barrier integrity. In this case, the combination of chronic oGVHD, sustained immunosuppression, and epithelial breakdown created a pathway for bacterial invasion. Paula A et al. found that corneal ulcers occurred 74.5 ± 54.3 months post-HSCT, with estimated 5- and 10-year incidence rates of 14% and 30%, respectively (11). Among these, 5%–10% may progress to endophthalmitis, posing a significant threat to vision. Epithelial disruption is a key factor commonly observed in oGVHD individuals. This disruption arises from the secretion of inflammatory cytokines such as interleukin-1Ra (IL-1RA), IL-6, IL-8, IL-10, IL-12p70, IL-17A, interferon-γ, and vascular endothelial growth factor, as well as collagen-degrading enzymes like matrix metalloproteinases 8 (MMP-8), matrix metalloproteinases 9 (MMP-9), and myeloperoxidase (MPO), which degrade the underlying extracellular matrix and cause tissue damage (12). Zeng et al. (13) analyzed 283 cases of endophthalmitis among 1,887 eyes with bacterial keratitis. In the BKH group (keratitis with hypopyon), *Staphylococcus epidermidis* accounted for the highest proportion (36.43%), while in the BKE group (endophthalmitis secondary to keratitis), it comprised 18.75%. These data confirm that *S. epidermidis* can progress from corneal infection to intraocular involvement through corneal damage. As an ocular surface commensal organism, *S. epidermidis* can colonize corneal ulcers and perforations, penetrating the cornea through compromised barrier integrity. Li et al. (14)demonstrated that corneal ulceration is a prerequisite for *S.epidermidis* corneal penetration. Patients with such presentations typically have delayed medical consultation (average 14.75 days), by which time corneal damage has progressed to deep stromal layers with disruption of barrier integrity, creating a “pathway” for bacterial invasion. As a conditional pathogen, *S. epidermidis* can colonize and perpetuate inflammation following corneal barrier breakdown, ultimately penetrating the intraocular space through ulcer perforation or stromal fissures. Ting and associates (15) compared culture-positive (n=128) and culture-negative (n=155) microbial keratitis cases and revealed that immunosuppression (caused by diabetes, systemic immunosuppressive treatment, malnutrition, or immunodeficiency) significantly increased the risk of microbial keratitis in individuals aged ≥50 years compared to those aged <50 years (26.1% vs 7.6%, P <0.01). The pathogen spectrum of oGVHD-related infectious keratitis and endophthalmitis has its unique characteristics. Using traditional culture-based methods, Shimizu and colleagues (16) observed significantly higher rates of positive cultures (75%) and multiple detections (positive culture for more than 2 species, 45.8%) among oGVHD cases compared to non-GVHD allo-HSCT cases (36% and 3.6%) and healthy subjects (25% and 0). They found *Staphylococcus Epidermidis*, α-hemolytic *Streptococcus*, Corynebacterium species, and Propionibacterium Acnes as the most common species among oGVHD subjects. Metagenomic next-generation sequencing has shown revolutionary advantages in the diagnosis of infectious diseases through unbiased broad-spectrum screening, rapid and accurate diagnosis, and the ability to identify mixed infections.

The clinical utility of mNGS in this case extended beyond mere pathogen detection. The patient had already received 3 days of empirical intravenous cefuroxime and topical broad-spectrum antibiotics without clinical improvement; the mNGS result exclusively identified *S. epidermidis* (a Gram-positive coagulase-negative staphylococcus), prompting a rational de-escalation from broad-spectrum empirical therapy to targeted intracameral, periocular, and systemic vancomycin. This sequence—empirical treatment failure → mNGS-directed pathogen identification → targeted antimicrobial switch → infection control with anatomical preservation—demonstrates the value of mNGS as a clinical decision-support tool rather than simply a research technique. In this case, our patient achieved pathogen identification through aqueous humor mNGS—a minimally invasive office-based procedure—without requiring vitrectomy.

Regarding treatment, vancomycin was selected for its reliable Gram-positive coverage. The intravitreal dose of 1 mg/0.1 mL follows established protocols, with therapeutic vitreous concentrations persisting for over 72 hours. In this case, infection was controlled without progression to corneal perforation, obviating the need for therapeutic vitrectomy. Amniotic membrane transplantation was performed after infection control was achieved, specifically to facilitate healing of the persistent corneal epithelial defect and relieve severe blepharospasm; it was not used as an anti-infective measure.

In summary, this case demonstrates that in immunosuppressed oGVHD patients with suspected endophthalmitis, aqueous humor mNGS can overcome the diagnostic limitations of conventional culture—particularly for commensal organisms such as *S. epidermidis*—and enable timely, targeted therapy while avoiding invasive surgical intervention.

### Limitation’s statement

This case report is inherently limited by its single-patient design and retrospective nature. While chronic immunosuppression is a recognized risk factor for opportunistic infections in allo-HSCT recipients, the critical teaching point of this case lies not in confirming this association, but in demonstrating how mNGS can overcome the diagnostic and therapeutic delay that often occurs when ocular surface commensals (e.g., *S. epidermidis*) are isolated from immunocompromised hosts. Conjunctival swab mgs was not performed; future studies may benefit from paired sampling of ocular surface and intraocular fluids to better delineate the route of infection. We acknowledge that the favorable anatomical outcome cannot be attributed to mNGS alone, but is the result of prompt AMT, multidisciplinary collaboration, and the patient’s preserved systemic resilience. Furthermore, the cost-effectiveness and optimal timing of mNGS relative to empirical therapy in oGVHD-related endophthalmitis remain undefined. Future prospective studies with larger sample sizes, standardized mNGS protocols, and multi-center collaborative data are warranted to establish whether routine mNGS screening of intraocular fluids should be incorporated into the diagnostic algorithm for oGVHD patients with suspected infectious keratitis progression, and to clarify the prognostic factors that distinguish salvageable eyes from those requiring vitrectomy or enucleation in this immunocompromised population.
